# Future climate forcing potentially without precedent in the last 420 million years

**DOI:** 10.1038/ncomms14845

**Published:** 2017-04-04

**Authors:** Gavin L. Foster, Dana L. Royer, Daniel J. Lunt

**Affiliations:** 1 Ocean and Earth Science, National Oceanography Centre Southampton, University of Southampton, Southampton SO14 3ZH, UK; 2 Department of Earth and Environmental Sciences, Wesleyan University, Middletown, Connecticut 06459, USA; 3 School of Geographical Sciences and Cabot Institute, University of Bristol, University Road, Bristol BS8 1SS, UK

## Abstract

The evolution of Earth's climate on geological timescales is largely driven by variations in the magnitude of total solar irradiance (TSI) and changes in the greenhouse gas content of the atmosphere. Here we show that the slow ∼50 Wm^−2^ increase in TSI over the last ∼420 million years (an increase of ∼9 Wm^−2^ of radiative forcing) was almost completely negated by a long-term decline in atmospheric CO_2_. This was likely due to the silicate weathering-negative feedback and the expansion of land plants that together ensured Earth's long-term habitability. Humanity's fossil-fuel use, if unabated, risks taking us, by the middle of the twenty-first century, to values of CO_2_ not seen since the early Eocene (50 million years ago). If CO_2_ continues to rise further into the twenty-third century, then the associated large increase in radiative forcing, and how the Earth system would respond, would likely be without geological precedent in the last half a billion years.

## Abstract (web summary)

Despite an increase in solar output, the Earth's climate has apparently remained relatively stable over geological time. Here, the authors compile atmospheric CO_2_ data for the past 420 million years and show that this climatic response is due to the long-term decline in this powerful greenhouse gas.

The primary source of energy in the Earth's climate system is incoming solar radiation, termed total solar irradiance, or TSI. At equilibrium, because of the requirement of energy conservation, the Earth's radiative budget must balance such that TSI is equal to outgoing longwave radiation at the top of the atmosphere (a state known as radiative equilibrium). Since the radiation emitted by a body is a function of surface temperature, the Earth's ‘effective temperature' (*T*_E_) is the temperature at which radiative equilibrium is achieved assuming the Earth acts like a blackbody, and can be calculated (in K) using the following expression:


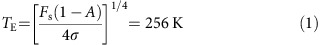


where *F*_s_ is the TSI (currently ∼1,368 Wm^−2^), *σ* is the Stefan–Boltzmann constant (5.67 × 10^−8^ W m^−2 ^K^−4^), *A*=0.29 (ref. 1) is the Earth's average planetary albedo (the fraction of incoming radiation scattered or reflected back out of the atmosphere by clouds, particulates in the atmosphere and the Earth's surface), and the factor of 4 accounts for the spherical and rotating nature of the Earth. The 31 K difference between *T*_E_ and the observed surface temperature of the Earth (+14.0 °C or 287.1 K is the 1961–1990 mean^2^) is almost entirely due to the action of the greenhouse effect (for example, ref. 3).

The majority (∼75%) of the greenhouse effect is due to the warming effects of water vapour and clouds, with the non-condensing greenhouse gasses (predominantly CO_2_ and CH_4_) accounting for the remaining 25% (ref. 3). However, at the temperatures and pressures typical of the Earth's surface, water vapour and clouds act as feedbacks rather than drivers of the greenhouse effect, with CO_2_ and CH_4_, and the other non-condensing GHGs (for example, N_2_O) determining the overall strength of the greenhouse effect^3^. Given this understanding, and that summarized in equation (1), the climatic evolution of the Earth over geological time is largely a function of the concentration of the non-condensing greenhouse gases, planetary albedo (A) and the TSI (*F*_s_; for example, ref. 4).

Owing to the way the Sun generates energy though the nuclear fusion of hydrogen into helium, over time its luminosity has increased^5^. This is a relatively well-understood process and the TSI at any time 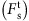 can be approximated by the following function^5^:


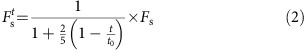


where *F*_s_ is the present day TSI (1,368 Wm^−2^), *t* is the time of interest since the formation of the Earth (Myrs) and *t*_0_ is the age of the Earth (4,567 Myrs (ref. 6), although here following ref. 5 we use a *t*_0_=4,700 Myrs). As a result, there has been an increase in TSI of ∼400 Wm^−2^ since the formation of the Earth. The following equation relates this change in TSI relative to today to radiative forcing (ΔF_sol_) assuming a constant planetary albedo (*A*) through time:


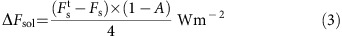


The global mean surface temperature change (Δ*T*, in K) for a given change in radiative forcing (Δ*F*, in Wm^−2^) can be described by:


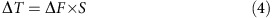


Where S is the sensitivity parameter^7^, in K W^−1^m^2^. Following ref. 4, and assuming an effective emissivity, *ɛ*, of the Earth of 0.6 (that is, 40% of longwave radiation is absorbed by greenhouse gases in the atmosphere), equation (1) can be used to define the following equation that describes *S* for today's climate (where the surface temperature=287.1 K) in the absence of any climate feedbacks (for example, water vapour, sea-ice etc.), also known as the Planck response (hence the subscript ‘P')^8^:


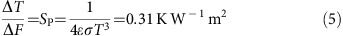


*S*_P_ depends on the overall strength of the greenhouse effect and the surface temperature of the Earth, and so this value is not necessarily applicable throughout Earth's history; nonetheless, this treatment provides a first-order constraint that the long-term secular increase in TSI of 400 Wm^−2^ would be associated with a secular warming of at least ∼20 K over the last 4.5 billion years, if albedo and emissivity remained constant. Since the combined effect of the greenhouse gas and other climate feedbacks (for example, water vapour, lapse rate, sea-ice etc.) is positive^8^, this is a minimum estimate of *S* and when all climate feedbacks are considered, *S*_a_ (where ‘a' denotes *actuo* after ref. 7) is likely in the range of 0.8– 1.6 K W^−1^ m^2^ (for example, refs 7, 9, 10, 11).

The actual evolution of Earth's temperature through geological time is a subject of considerable debate (for example, ref. 12), yet there is a longstanding view that, despite the increase in solar output, Earth's surface temperature was, for much of geological time, warmer, not colder, than today (for example, refs 13, 14). This apparent inconsistency is known as the ‘Faint Young Sun' paradox and to reconcile the observation of a relatively stable climate in the face of increasing solar output through time requires a parallel change in some other factors that influence Earth's radiative budget. First and foremost among these factors is thought to be a concomitant reduction in the strength of the greenhouse effect, and in atmospheric CO_2_ concentration in particular (for example, refs 15, 16). The radiative forcing from changing CO_2_ can be estimated using the following formulation^17^:


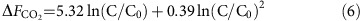


where Δ*F*_CO_2__ is the radiative forcing from CO_2_ change (in Wm^−2^), *C* represents the concentration of CO_2_ at the time of interest and *C*_0_ is the pre-industrial concentration of CO_2_=278 p.p.m. For the decrease in atmospheric CO_2_ to balance the increase in solar output over time, the following equation is required to be true (or at least approximately so):


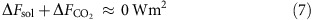


In this formulation we are ignoring the other non-condensing greenhouse gases (for example, CH_4_, N_2_O). This is partly out of practical necessity since there are currently no proxies for these gases, but is justifiable to a first order because variations in CO_2_ accounted for ∼80% of the greenhouse gas forcing on glacial–interglacial timescales^4^ and CO_2_ was likely the dominant greenhouse gas for the past 500 Myrs (refs 18, 19, 20).

Testing whether the apparent stability of surface temperature is a consequence of the relative stability of climate forcing, therefore, requires high-density, high-quality data on past atmospheric CO_2_ levels. Consequently, in this study we compile the available CO_2_ data for the last 420 Myrs where, although the magnitude in the change of solar output is reduced relative to changes on longer timescales, data availability is sufficient to provide a detailed picture of the evolution of atmospheric CO_2_. The relative stability of climate over this interval is constrained by three lines of evidence: (i) the continued presence of complex life, given the thermal limits of metazoans (<40–50 °C; ref. 21) and despite a number of mass extinctions^22^; (ii) the continuous presence of liquid water and the occurrence of climate-sensitive sedimentary deposits (for example, coal versus glacial deposits) that indicate warm and cold periods but no evidence of an overall long-term secular trend (Fig. 1)^23^; and (iii) moderate tropical sea surface temperatures (25–40 °C) as recorded by oxygen and clumped isotope analysis of fossil marine carbonates^24^,^25^,^26^,^27^. Our new compilation shows that this climate stability was the result of a long-term decline in atmospheric CO_2_ that, in terms of radiative forcing, approximately cancelled out the increase in solar output. This long-term view of climate forcing provides a valuable geological context for potential levels of CO_2_ in our warming future.

## Results

### A new CO_2_ compilation

In order to better understand the role of the greenhouse effect in Earth's climate evolution, we have compiled ∼1,500 discrete estimates of atmospheric CO_2_ from five independent techniques drawn from 112 published studies covering the last 420 Myrs (Supplementary Data 1). We uniformly apply a set of criteria and the latest understanding, described in the supplement, to screen the available CO_2_ records. By following these criteria, the ages and CO_2_ values associated with some records are revised (see Methods), and ∼1/5 of the published estimates have been excluded (leaving *n*=1,241 in our final compilation). This standardization process helps ensure the highest-quality compilation whose individual records can be more cleanly compared to one another.

**Figure 1:**
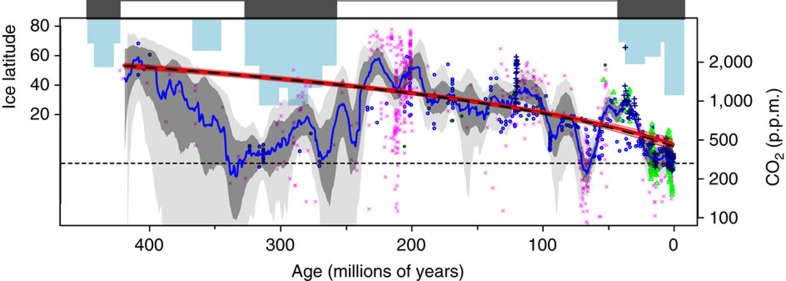
Temporal evolution of climate and atmospheric CO_2_. Latitudinal extent of continental ice deposits^23^ (blue bars) and multi-proxy atmospheric CO_2_ (in p.p.m.) compiled from the literature (data found in Supplementary Data 1; symbols). CO_2_ from leaf stomata shown in blue circles, pedogenic carbonate δ^13^C as pink crosses, boron isotopes in foraminifera as green triangles, liverwort δ^13^C as dark blue filled circles and δ^13^C of alkenones as dark blue crosses. The most likely LOESS fit through the data, taking into account X- and Y- uncertainty is shown as the blue line (data found in Supplementary Data 2). 68 and 95% confidence intervals are shown as dark and light grey bands. Red line is a linear best fit (curved due to log-scale for *y* axis) and 95% confidence interval for least squares regression through the CO_2_ data (*m*=3.4±0.17 1 s.e.m., *R*^2^=0.26, *P*<0.0005). Black line is least squares fit through the LOESS best fit (blue line) resampled to original data density (*m*=3.5±0.12 1 s.e.m., *R*^2^=0.44, *P*<0.0005). Dashed line is pre-industrial CO_2_ (278 p.p.m.). Icehouse time intervals are indicated by a black band and greenhouse intervals by a white band.

Our new compilation is shown in Fig. 1 and, compared to older compilations, there is better agreement between the different methods of CO_2_ reconstruction^28^. In this case, this is largely due to the refinement of the pedogenic carbonate proxy following ref. 29 (see Methods). However, despite our efforts to improve the compilation, there are still relatively large uncertainties at times and disagreements remain between techniques for some time intervals (Fig. 1). It is also apparent that the early parts of the record rely on fewer observations and are characterized by a reduced diversity in proxy type (Supplementary Fig. 1). In order to gain a better appreciation of the multi-million-year evolution of CO_2_, and in light of these uncertainties and limitations we have followed a probabilistic approach where Monte Carlo resampling is used to generate 1,000 artificial time series of CO_2_ with each data point randomly perturbed within its age (*X*) and CO_2_ (*Y*) uncertainty. Each realization was then interpolated to a regular 0.5 Myr spacing and a LOESS fit was performed with the degree of smoothing optimized by generalized cross-validation^30^. At each time step the distribution of LOESS fits was evaluated and the maximum probability, hence the most likely value for long-term CO_2_, and the associated 68 and 95 percentile ranges were determined (Fig. 1 and Supplementary Data 2).

Our new compilation and probabilistic treatment reveals that for the last ∼420 Myrs, CO_2_, on the whole, has been elevated compared to pre-industrial values (278 p.p.m.). The highest CO_2_ values of ∼2,000 p.p.m. were reached during the Devonian (∼400 Myrs ago) and Triassic (220–200 Myrs ago), with individual estimates ranging up to a maximum of ∼3,700±1,600 p.p.m. at 215 Myrs. In contrast, values close to pre-industrial are found during much of the Carboniferous (∼300 Myrs ago) and late Cretaceous (∼80 Myrs ago). A linear fit to either the entire CO_2_ compilation, or a resampling of our LOESS fit to reflect the original data density, reveals that long-term average CO_2_ has declined over the last 420 Myrs by ∼3.4 p.p.m. per Myrs (Fig. 1).

### Radiative forcing through the last 420 million years

**Figure 2:**
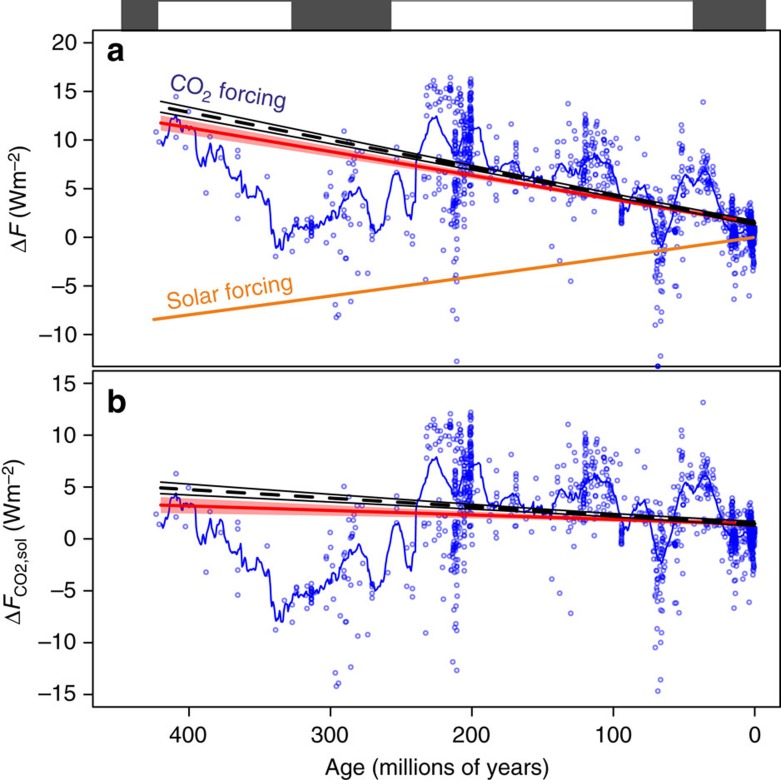
Temporal evolution of climate forcing. (**a**) CO_2_ data (blue circles) and LOESS best fit (blue line; Supplementary Data 2) with Δ*F* for CO_2_ calculated as in equation (6). The change in solar forcing (Δ*F*_sol_) calculated using equations (2 and 3). (**b**) Δ*F*_CO2,sol_ for data (blue circles) and LOESS best fit (blue line). Red line is a linear best fit and 95% confidence interval for least squares regression through the Δ*F*_CO2,sol_ data (blue circles; *m*=−0.004±0.001 1 s.e.m., *R*^2^=0.01, *P*=0.0003). Black line is least squares fit through the LOESS best fit resampled to original data density (blue line; *m*=0.008±0.001 1 s.e.m., *R*^2^=0.06, *P*<0.0001). Icehouse time intervals are indicated by a black band and greenhouse intervals by a white band.

**Figure 3:**
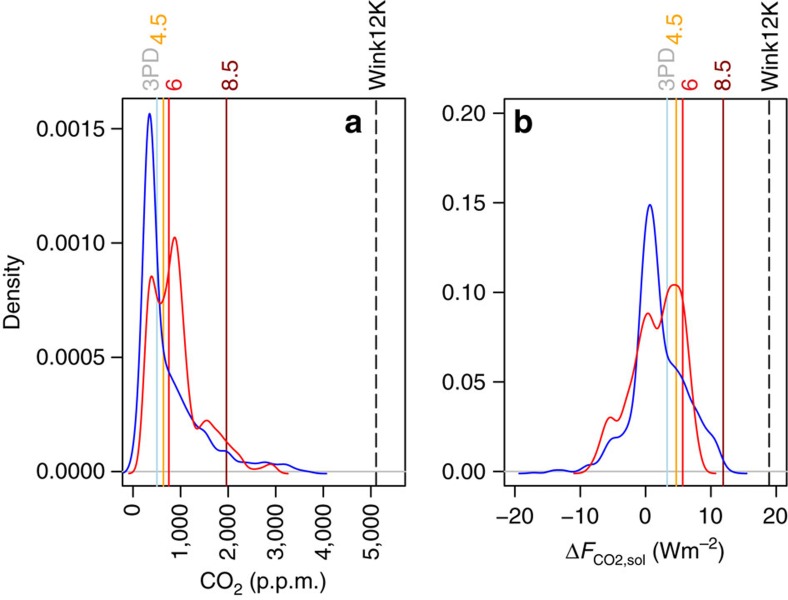
Probability density functions of climate forcing. (**a**) Atmospheric CO_2_ with proxy data in blue (Supplementary Data 1) and LOESS best fit (Supplementary Data 2) in red. Vertical lines show maximum atmospheric CO_2_ from relevant representative concentration pathways (RCP) (colours)^39^ and from a 12,000 Pg C scenario (from ref. 41; Wink12K, black dashed line). (**c**) Proxy-based Δ*F*_CO2,sol_ in blue and LOESS best fit in red along with relevant RCP scenarios (colours)^39^ and 12,000 Pg C scenario (black dashed line)^41^.

Radiative forcing from CO_2_ change can be calculated using equation (6). This can then be combined with the radiative forcing from the increase in solar output defined by equation (3) (Fig. 2a); the temporal evolution of the simple sum of these terms (Δ*F*_CO2,sol_) is shown in Fig. 2b. As has been noted elsewhere with prior Phanerozoic CO_2_ compilations (for example, refs 31, 32), there is a good first-order agreement between CO_2_ levels and the occurrence of greenhouse/icehouse states (Fig. 1): CO_2_ is high during greenhouse climate states and low during icehouse climates. Our data support previous work suggesting that, when Δ*F*_CO2,sol_ is considered, icehouse states generally occur when climate forcing drops below approximately +1–2 Wm^−2^ (refs 31, 32). This treatment also reveals that for the last 420 Myrs, despite a gradual 4% increase in solar luminosity (equivalent to +9 Wm^−2^ of radiative forcing), there has been very little long-term change in Δ*F*_CO2,sol_ (despite shorter-term fluctuations of up to 10 Wm^−2^), with a linear fit to all the data suggesting only a slight decrease of 0.004 Wm^−2^ per Myrs (±0.001 1 s.e.m.; *P*=0.0003; red line on Fig. 2b); a linear fit to our LOESS fit resampled with the original data density yields a slightly higher negative slope (−0.008±0.001 1 s.e.m.; *P*<0.0001; black line Fig. 2b). This indicates that the long-term decrease in CO_2_ over the last 420 Myrs has largely compensated for the increase in solar output over the interval. In terms of shorter-term radiative imbalances, 95% of the Δ*F*_CO2,sol_ values are within ±7 Wm^−2^ and 68% within +5/−3 Wm^−2^ (Fig. 3). Thus, even on multi-million-year timescales (and sometimes shorter) the external radiative forcing (not considering the action of additional feedbacks internal to the climate system such as the ice-albedo feedback) has not varied beyond ±2.0% of the total incoming radiation (∼340 Wm^−2^).

## Discussion

The concentration of atmospheric CO_2_ on multi-million-year timescales depends largely on the balance between the input of CO_2_ from volcanism, metamorphism and organic carbon weathering and the output of CO_2_ from silicate weathering and organic carbon burial^15^. Since the magnitude of silicate weathering is climatically sensitive (that is, a function of temperature and precipitation; for example, ref. 33), silicate weathering also buffers the CO_2_ content of the atmosphere by acting as a negative feedback^15^. However, we note that in many modern settings silicate weathering is supply-limited^33^. Thus, for silicate weathering to operate as a climate feedback, the Earth must be sufficiently tectonically active to ensure an adequate supply of fresh minerals^34^.

The marked ‘double-hump' pattern of the CO_2_ reconstruction (Fig. 1) that is also common to other compilations (for example, refs 28, 35) is likely caused by changes in the inputs and outputs of CO_2_ in response to the supercontinent cycle^36^. For example, the low CO_2_ during the Carboniferous (∼300 Myrs ago) and during the later parts of the Cenozoic (last 65 Myrs) was likely a result of a reduced volcanic flux and/or enhanced silicate weathering, at least in part due to higher continental relief during supercontinent construction phase. During the intervening greenhouse intervals the reverse was likely true (high volcanic flux and/or low silicate weathering due to low relief)^36^,^37^. An additional factor in the decline in CO_2_ was the overall enhancement of silicate weathering due to the expansion of the terrestrial biosphere over the last 400 million years or so (refs 16, 38). It is probably this expansion, along with the silicate weathering-negative feedback (facilitated by sufficiently active plate tectonic regime^34^), that was the key in keeping Δ*F*_CO2,Sol_ relatively constant over the long term (for example, ref. 16). Indeed, as with previous compilations^35^, our new CO_2_ record largely agrees with the GEOCARB carbon cycle model that incorporates these long-term changes in silicate weathering (Supplementary Fig. 2), especially when the uncertainties in both records are considered^35^. This serves to further underscore the importance of the rise of the terrestrial biosphere, along with plate tectonics and silicate weathering, in ensuring climate stability through time^16^ and provides additional support for our broad understanding of the Earth's long-term carbon cycle as encapsulated by the GEOCARB model^35^,^38^ (Supplementary Fig. 2).

**Figure 4:**
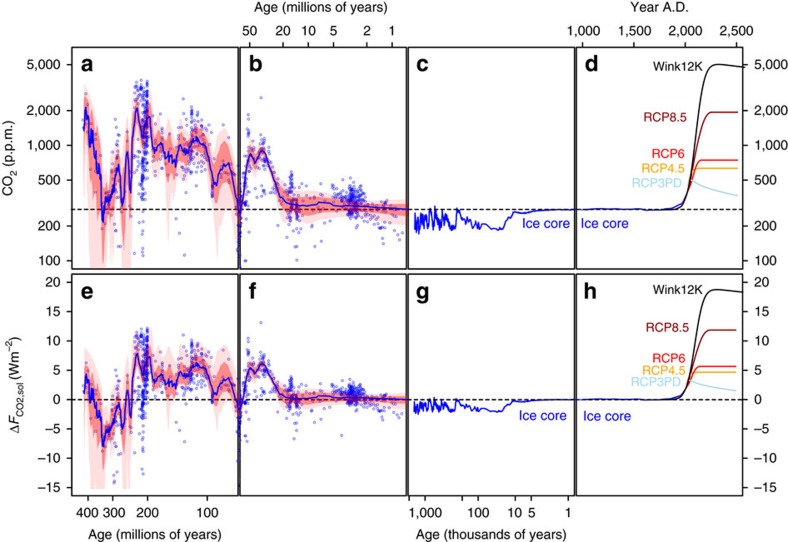
Temporal evolution of CO_2_ and climate forcing. (**a**,**b**) Proxy-based atmospheric CO_2_ (Supplementary Data 1) on a log timescale with best fit LOESS and associated uncertainty envelope (Supplementary Data 2). (**c**) Ice core atmospheric CO_2_ from ref. 82 on log timescale. (**d**) Atmospheric CO_2_ on line timescale from ice core and observation record^82^ and future RCP^8^ and other^41^ scenarios (RCP3PD—grey, RCP4.5—orange, RCP6—red, RCP8.5—brown, Wink12k -black)^38^,^40^. (**e**–**h**) Δ*F*_CO2,sol_ calculated from data shown in **a**–**d** as described in text. In (**c**) Δ*F*_CO2,sol_ is calculated from ice core CO_2_ estimates assuming no change in solar output^82^. No change in solar output is also applied to the records in **h**.

Regardless of the ultimate cause for the observed relative stability in Δ*F*_CO2,sol_ over the last 420 million years, business-as-usual emission scenarios (for example, representative concentration pathway RCP8.5)^39^ for fossil fuel emissions suggest that atmospheric CO_2_ could peak in 2,250 AD at ∼2,000 p.p.m. CO_2_ values as high as this were last seen in the Triassic around 220–200 Myrs ago (Figs 3 and 4). However, because of the steady increase in solar output over time, in terms of radiative forcing by the end of this century RCP8.5 is similar to the early Eocene, and by 2,250 AD exceeds what is recorded in the geological record for at least 99.9% of the last 420 Myrs (Figs 3 and 4). A recent study suggested that if both conventional and non-conventional fossil fuel reserves (amounting to ∼12,000 Pg C; ref. 40) were exhausted in such a business-as-usual scenario, atmospheric CO_2_ could rise to ∼5,000 p.p.m. by 2,400 AD (refs 41, 42), which is clearly higher, in terms of both forcing and absolute CO_2_, than at any time captured by our compilation (Figs 3 and 4, Wink12K scenario). Such a scenario therefore risks subjecting the Earth to a climate forcing that has no apparent geological precedent, for at least the last 420 Myrs. We should be aware of course of the limitations of the geological record, and it is debatable whether an extreme climate change event analogous to the Anthropocene, if it existed at all, would leave a detectable signal, given our current CO_2_ proxies and records^43^. Nonetheless, prolonged warm greenhouse climate states have occurred in the past, but the rates of climate change in the geological record are on the whole very likely slower than what we are currently experiencing (for example, ref. 44). Unabated fossil fuel use therefore has the potential to push the climate system into a state that has not been seen on Earth in at least the last 420 Myrs.

## Methods

### Assembling the CO_2_ compilation

The new CO_2_ compilation here consists of 1,241 independent estimates coming from five proxy methods and 112 published studies. In putting this compilation together, we applied a uniform set of criteria described next to screen potential CO_2_ records. By following these criteria, the CO_2_ values associated with some records are revised, while other records are excluded all together. This standardization process helps ensure a high-quality compilation whose individual records can be more cleanly compared to one another. Of course, some of our criteria may be judged at some point to be incorrect. It is in this light, and in the spirit of full transparency, that we describe our criteria. We consider our compilation to be a ‘living document', not only because in the future new records will be added but because existing records will be interpreted in new ways. The compilation can be found in Supplementary Data 1.

First, we excluded all goethite-based CO_2_ estimates^45^,^46^,^47^,^48^ due to uncertainties in modelling some of the isotopic fractionation factors^49^. We also excluded all B/Ca-based estimates^50^ because the environmental controls on B/Ca are currently not well understood^51^. The nahcolite proxy is based on well-described mineral phase equilibria^52^, but we exclude the published estimates because it is not possible to assign mean values (all values within a defined CO_2_ interval are equally probable). It is important to note, however, that the range described by these nahcolite estimates agree well with the other data in our compilation. We do not include the boron-based estimates of Pearson and Palmer^53^ owing to problems related to potential diagenesis, potential analytical issues, vital effects of extinct species and the evolution of seawater δ^11^B and alkalinity (as discussed in ref. 19). For the alkenone-based records of Pagani and colleagues^54^,^55^,^56^,^57^,^58^, we adopt the compilation of ref. 58 who imposed a uniform set of quality controls on the original data sets and used a TEX_86_ temperature record (versus the δ^18^O of ref. 54). We update all Cenozoic liverwort-based estimates^59^ with the atmospheric δ^13^C model of ref. 60.

For estimates calculated from stomatal ratios (SR, where SR=ratio of stomatal frequency in nearest living equivalent to fossil), two transfer functions to CO_2_ have been proposed: 1 SR=1 RCO_2_ and 1 SR=2 RCO_2_, where RCO_2_=ratio of atmospheric CO_2_ in the past to pre-industrial conditions (300 p.p.m.; refs 61, 62). Following Beerling and Royer^63^, we use these two functions to establish the uncertainty bounds in estimated CO_2_. Owing to the *ad hoc* nature of these functions, CO_2_ estimates from stomatal ratios should be considered semiquantitative only. We modify many of the stomatal-based estimates of Retallack^64^. First, we only use estimates associated with five or more cuticle fragments, as this is the minimum sampling required in most cases for robust estimates^65^. We use the quantitative transfer function of Royer^65^ for estimates coming from *Ginkgo adiantoides*, the fossil species considered conspecific with extant *G. biloba*^66^. For estimates coming from other species within the *Ginkgo* genus we use the stomatal ratio approach, and we exclude estimates not coming from *Ginkgo* as these other groups are too distantly related to *Ginkgo* and have not been calibrated correctly^67^.

A recent development that we have incorporated is the lowering of CO_2_ estimates from the pedogenic carbonate method^29^. This is because one of the key input parameters, the concentration of biologically derived CO_2_ in the soil (*S*(z)), had been overestimated in most paleosols by a factor of two or more^29^,^68^. Here we adjust most CO_2_ estimates assuming a *S*(z) of 2,000 p.p.m. (ref. 68). This is a simple correction that masks variability in *S*(z)^69^, but it is nonetheless a useful place to start and in most cases represents an improvement over previous estimates^70^. This is an area where we anticipate substantial revision in the near future. Along these lines, several proxies for *S*(z) have been proposed^69^,^70^,^71^,^72^,^73^,^74^,^75^,^76^, and we use estimates of *S*(z) derived from these methods when available. We also replace some of the pedogenic carbonate-based estimates of ref. 73 with those based on the same sediments^74^,^75^ because these newer studies provide better constraints on organic matter δ^13^C. Lastly, we collapse the high-resolution stomatal-based record of ref. 76 into one estimate calculated from the mean of the individual estimates.

Uncertainties in paleo-CO_2_ estimates have been assessed in different ways. Most earlier uncertainties associated with the pedogenic carbonate method, for example, only incorporate uncertainty in *S*(z); potential uncertainties in other input variables are ignored. In addition, errors associated with stomatal-based methods that involve quantitative regression equations usually only reflect uncertainty in the regressions; uncertainties in the fossil measurements are ignored. This means that error bars can convey different meanings, which hinders comparisons across methods. In recent years, Monte Carlo simulations for propagating uncertainty in multiple input variables have been developed for all five proxy systems^11^,^55^,^59^,^77^. So long as the reported percentile ranges are identical, these simulations facilitate better cross-method comparisons.

We attempt here to standardize reported uncertainties so that they incorporate uncertainty in all input variables; we also standardize the percentile levels (16 and 84; equivalent to ±1 s.d. for a normally distributed population). For uncertainties derived from Monte Carlo simulations that propagate uncertainties in most or all input variables (this covers most estimates from the alkenone, boron and liverwort proxies, as well as most recent estimates from pedogenic carbonates and stomata), we adjust if needed to the 16 and 84 percentile levels: for example, if the 2.5 and 97.5 percentiles are reported, we assume the population has a normal distribution and cut the uncertainties in half. Because most paleo-CO_2_ estimates have a right-skewed distribution, this means that we are overestimating the magnitude of the upper uncertainty and underestimating the magnitude of the lower uncertainty. For uncertainties with the pedogenic carbonate method derived by varying only *S*(z), we adopt for the 16 and 84 percentiles the generic recommendation of +100%/−50% of the median value calculated from multifactorial Monte Carlo simulations^78^; these uncertainties are larger and probably more representative than the reported uncertainties. Similarly, for stomatal estimates whose uncertainties only take into account variance in the transfer function, we assume 16 and 84 percentiles equal to +100%/−40% of the median value, a range that takes into account representative uncertainty in both the transfer function and the fossil measurements^77^.

All ages have been updated to the 2012 Geologic Time Scale^79^. Many CO_2_ estimates from terrestrial proxies have biostratigraphic (for example, ‘Maastrichtian'), magnetostratigraphic or chronostratigraphic constraints. For records with biostratigraphic or magnetostratigraphic constraints, we update their ages following the new Time Scale. Many CO_2_ estimates have no reported age uncertainties. In almost all cases, these estimates have excellent age control, the most common example being estimates from marine sediment cores (for example, all boron- and alkenone-based estimates). Some terrestrial-based data fall under this category too, for example, the Triassic–Jurassic sediments in the Newark Supergroup^80^. When no age uncertainty is reported we take the 1 s.d. to be 0.001 Myr for marine records and 4% for terrestrial records.

### Monte Carlo sampling and LOESS fitting

Monte Carlo approach was used to generate 1,000 realizations of the multiproxy CO_2_ time series with each data point randomly perturbed within its age and CO_2_ uncertainty as given in Supplementary Data 1. For CO_2_ estimates with asymmetric uncertainties, we force symmetry by assuming ±1 s.d. is equal to the average offset between the median and the 16 and 84 percentiles. Each realization of the time series was then interpolated to a regular 0.5 Myr spacing and LOESS curves were fitted to each interpolated realization using the programme R^81^. The optimal degree of smoothing was determined for each interpolated realization using generalized cross-validation^30^. At each 0.5 Myr time step the distribution of LOESS curves was evaluated and the probability distribution was determined. From this probability distribution the most likely value (or probability maximum) and the upper and lower limits corresponding to 68 and 95% confidence limits were identified. These variables are given in Supplementary Data 2.

Owing to the nature of the proxies used the make-up and coverage of our new CO_2_ compilation varies considerably as a function of time. By grouping the data into 2.5 million year bins, Supplementary Fig. 1 shows that the number of observations in each 2.5 million year bin generally increases towards the present, with a notable exception of a peak at ∼200 million years ago at the Triassic–Jurassic boundary. Supplementary Fig. 1 also shows that the boron isotope and alkenone δ^13^C methods are restricted to the Cenozoic and liverwort δ^13^C proxy are concentrated in the middle part of the last 420 million years. Estimates based on fossil plant stomata appear focused in the last 200 million years, with only sporadic coverage over the early part of the interval. Only CO_2_ estimates from pedogenic carbonate δ^13^C method are relatively evenly spread across the last 420 million years. To examine the influence of this uneven distribution of proxy types we performed the LOESS fitting a further two times but without one of the dominant proxy systems (no-pedogenic carbonate, no-stomata), these alternatives are shown with the complete data set in Supplementary Fig. 3. While subtle variations exist, overall the large-scale structure remains intact, although if the pedogenic carbonate estimates are discarded the early part of the Phanerozoic is largely under sampled. In terms of long-term CO_2_ change, however, discarding all the estimates from either the pedogenic carbonate method or the stomatal method does not change our principal conclusions. For instance, the long-term CO_2_ decline over the last 420 million years without the pedogenic carbonates is 2 p.p.m. per million years, and without the estimates from the plant stomata method it is 4 p.p.m. per million years. Both of which are similar to the CO_2_ decline we determine for the complete data set (3.4 p.p.m. per million years), indicating that a long-term decline in CO_2_ over the last 420 million years is a robust observation. In terms of Δ*F*_CO2,sol_ the long-term trends are also similar being 0.008±0.001 Wm^−2^ (*R*^2^=0.03 and *P*<0.001) and 0.0001±0.001 Wm^−2^ (*R*^2^=<0.001, *P*=0.94) for no-stomata and no-pedogenic carbonate, respectively.

### Data availability

The authors declare that all data supporting the findings of this study are available within the Supplementary Information files associated with this manuscript.

## Additional information

**How to cite this article:** Foster, G. L. *et al*. Future climate forcing potentially without precedent in the last 420 million years. *Nat. Commun.* **8,** 14845 doi: 10.1038/ncomms14845 (2017).

**Publisher's note**: Springer Nature remains neutral with regard to jurisdictional claims in published maps and institutional affiliations.

## Supplementary Material

Supplementary Information Supplementary Figures

Supplementary Data 1 Phanerozoic CO2 compilation used in analyses

Supplementary Data 2 LOESS fit to the CO2 data set in Sup. Data 1.
