# Supplementary material for: Future climate forcing potentially without precedent in the last 420 million years
Source: Nat Commun. 2017 Apr 4;8:14845. doi: 10.1038/ncomms14845 (PMC5382278; doi:10.1038/ncomms14845)
Supplement: Supplementary Information — Supplementary Figures [file ncomms14845-s1.pdf]

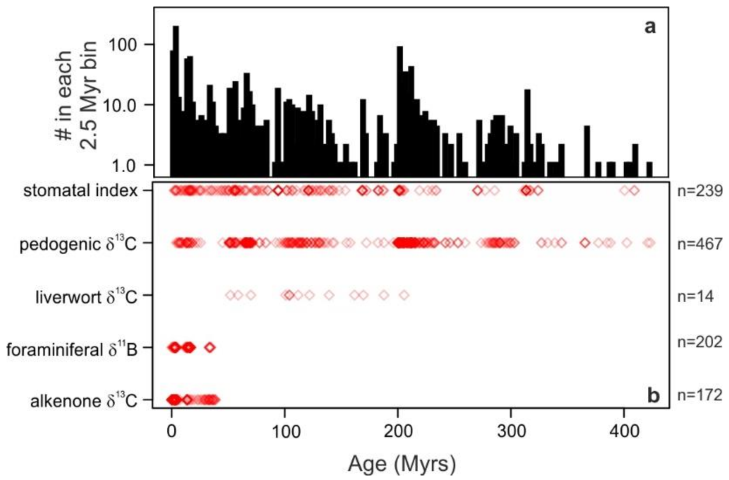


**Supplementary Figure 1. Distribution of $CO_2$ observations over the last 420 million years.** (a) Number of observations in each 2.5 million year bin, note the log-scale. (b) Distribution of $CO_2$ observations by proxy type, n is the number of observations.

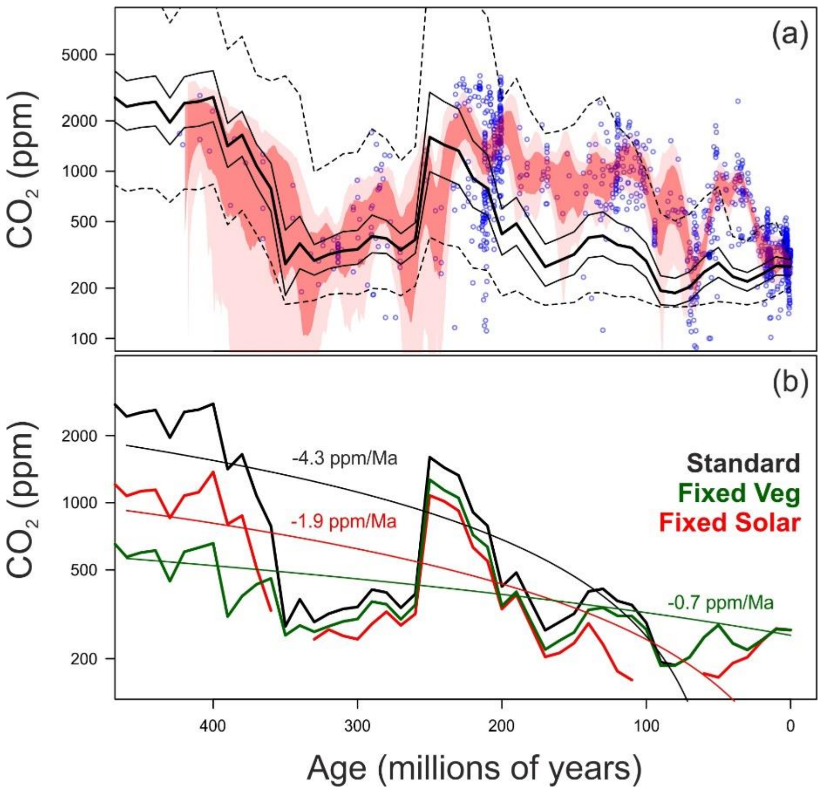


**Supplementary Figure 2. Comparison of $CO_2$ with GEOCARB model and a sensitivity analysis. (a)** The $CO_2$ compilation produced here (shown as open blue circles and LOESS fit – dark red for 68% confidence band and light red for 95% confidence band; this is identical to Figure 1) compared to $CO_2$ estimates from the GEOCARB long-term carbon cycle model[36]. Because some of the inputs in the GEOCARB model are poorly constrained, it is difficult to establish quantitative, fully-propagated uncertainties; its true uncertainties—at 95% confidence—probably lie between the two envelopes shown (dotted and solid lines) (see ref. 36 for justification). (b) An exploration in the GEOCARB standard model of the effect of fixing terrestrial vegetation cover (green) and solar luminosity (red) at modern values. Best fit lines and associated gradients are shown with the appropriate colour. This sensitivity analysis shows that in the GEOCARB model, the enhancement of silicate weathering in response to the expansion of terrestrial vegetation at ~350 million years ago[36] along with the increase in solar luminosity were key factors in the decline in atmospheric $CO_2$ during the Phanerozoic.

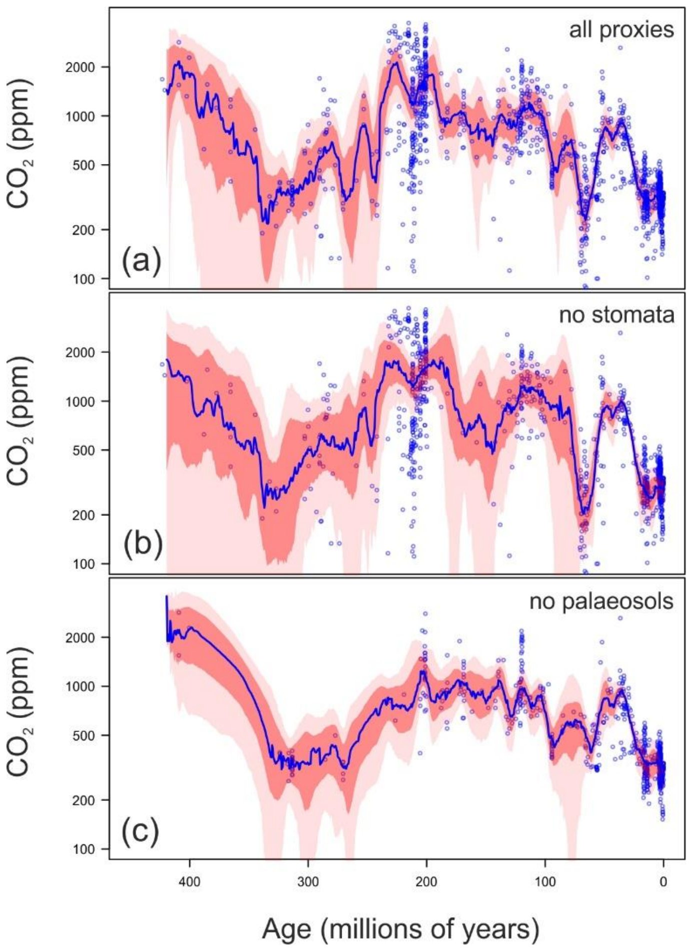


**Supplementary Figure 3. Evolution of atmospheric $CO_2$ over the last 420 million years.** (a) All proxy records as shown in Figure 1. (b) $CO_2$ over the last 420 million years excluding all stomatal density estimates. (c) $CO_2$ over the last 420 million years excluding all pedogenic carbonate estimates.
